# Research on Comprehensive Vehicle Information Detection Technology Based on Single-Point Laser Ranging

**DOI:** 10.3390/s25051303

**Published:** 2025-02-20

**Authors:** Haiyu Chen, Xin Wen, Yunbo Liu, Hui Zhang

**Affiliations:** Guangdong Provincial Key Laboratory of Intelligent Transportation System, School of Intelligent Systems Engineering, Shenzhen Campus, Sun Yat-sen University, Shenzhen 518107, China; chenhy253@mail2.sysu.edu.cn (H.C.); a380003220@outlook.com (X.W.); liuyb68@mail2.sysu.edu.cn (Y.L.)

**Keywords:** intelligent roads, vehicle detection, sensing technologies, single-point laser ranging, adaptive threshold state machine

## Abstract

In response to the limitations of existing vehicle detection technologies when applied to distributed sensor networks for road traffic holographic perception, this paper proposes a vehicle information detection technology based on single-point laser ranging. The system uses two single-point laser radars with fixed angles, combined with an adaptive threshold state machine and waveform segmentation fusion, to accurately detect vehicle speed, lane position, and other parameters. Compared with traditional methods, this technology offers advantages such as richer detection dimensions, low cost, and ease of installation and maintenance, making it suitable for large-scale traffic monitoring on secondary roads, highways, and suburban roads. Experimental results show that the system achieves high accuracy and reliability in low-to-medium-traffic flow scenarios, demonstrating its potential for intelligent road traffic applications.

## 1. Introduction

In recent years, the continuous development of Intelligent Transportation Systems (ITSs) has placed higher demands on the traffic perception capabilities of smart roads, especially in terms of real-time performance, accuracy, and comprehensive sensing. However, traditional vehicle detection technologies are limited by their detection principles and data collection ranges, making it difficult to achieve comprehensive traffic condition awareness based on bayonet detection methods. For instance, a single sensor (such as an induction loop or geomagnetic detector) can only capture information about the presence of vehicles in a localized section and lacks the ability to continuously track parameters like vehicle speed, type, and travel trajectory. Furthermore, it cannot construct a dynamic traffic flow model for the entire road cross-section. This point-based, discrete data collection mode makes it challenging to support the real-time, full-scale traffic situation analysis required by smart roads, thus becoming a key bottleneck limiting the implementation of advanced applications like vehicle–road collaboration and autonomous driving.

With the gradual maturity of multi-sensor fusion technology, achieving large-scale and accurate traffic perception through sensor networks [1,2,3,4] or radar–vision integrated fusion [5,6] has become a research hotspot in the field of intelligent road traffic perception. However, despite the ability of radar–vision integrated fusion technology to provide large-scale and accurate traffic perception, it faces challenges such as high equipment costs, complex installation, difficult maintenance, and reliance on high-performance computing devices. For economically underdeveloped regions, limited funding makes it difficult to bear such a large investment requirement. Moreover, in traffic scenarios such as secondary roads with low-to-medium traffic, mountainous highways, and suburban roads, the application of radar–vision integrated technology can result in redundant monitoring capabilities, which do not match the cost–benefit ratio. Deploying radar–vision integrated devices for large-scale traffic monitoring is not economically viable. In contrast, sensor networks, which are low-cost and highly expandable, are more suitable.

Currently, in the field of road traffic perception, commonly used vehicle detection technologies for wireless sensor networks [7,8,9,10] include inductive loop detection, geomagnetic detection, ultrasonic detection, video detection, and LiDAR detection. However, these vehicle detection technologies are limited by their detection principles and implementation methods, resulting in issues such as complex installation, difficult operation and maintenance, and high costs when deployed on a large scale. For example, inductive loop detection [11,12,13] requires cutting the road surface during installation, which can affect the road’s lifespan. It also has low flexibility and is difficult to maintain. Geomagnetic detection technology [14,15,16,17,18] struggles to detect stationary or low-speed vehicles, and its materials are prone to aging, with sensitivity decreasing year by year, requiring regular calibration and maintenance. Ultrasonic technology [19,20,21] and video detection technology [22,23,24,25,26] are easily influenced by surrounding environmental factors, have difficult installation and maintenance, and come with high deployment costs. LiDAR detection technology [27,28,29,30,31] is expensive, with large equipment, and complex installation and maintenance processes. As a result, the coverage rate of vehicle detection devices in current road traffic networks remains relatively low, with significant uneven distribution. Existing vehicle detection devices are mostly applied in major urban roads and highways, while regions such as secondary roads with low traffic, mountainous highways, and suburban roads have a noticeable lack of detection device coverage, severely limiting the realization of comprehensive traffic perception.

In addition, as shown in Table 1, inductive loop detection, geomagnetic detection, and ultrasonic detection technologies have relatively simple detection dimensions, detecting only one lane. When constructing sensor networks, these technologies often require high deployment density. Specifically, when measuring vehicle speed, inductive loop detection and geomagnetic detection typically require multiple sensors to be used together, which increases the difficulty and workload in later operation and maintenance.

To address the above issues, this paper proposes a vehicle comprehensive information detection technology based on single-point laser ranging. Two single-point laser ranging radars, fixed at a certain angle, are used to continuously monitor changes in object distance signals within a section. The adaptive threshold state machine detection algorithm is then applied to preliminarily extract valid waveforms. These waveforms are further segmented and fused to extract vehicle information such as speed and the lane in which the vehicle is traveling. As shown in Table 1, compared with existing vehicle detection technologies, this technology offers advantages such as rich detection dimensions, easy installation and maintenance, and low cost. It is suitable for large-scale traffic monitoring in secondary roads with low-to-medium traffic, mountainous highways, and suburban roads.

The contributions of this study are as follows.

(1) The development of a low-cost, easy-to-deploy roadside vehicle detection system: This paper designs and implements a vehicle detection system based on single-point laser ranging. By using two fixed-angle single-point laser range radars, combined with an adaptive threshold state machine detection algorithm, and waveform segmentation and fusion processing methods, the system achieves vehicle recognition, lane discrimination, and speed estimation. Compared with traditional vehicle detectors, this solution is cost-effective, simple in structure, and facilitates rapid roadside deployment. It can serve as a distributed multi-point vehicle detector, suitable for low-traffic and moderate-traffic road environments.

(2) The proposal of a vehicle detection and speed estimation algorithm based on occlusion judgment: This study proposes a detection algorithm that can handle the effects of occlusion. By distinguishing between front and rear occlusions, the algorithm improves vehicle detection accuracy and speed estimation precision to some extent. Experimental results show that this algorithm demonstrates high detection accuracy in low-traffic environments and successfully achieves the accurate identification of multiple vehicles even under occlusion effects in high-traffic environments.

## 2. System Description

**Figure 1:**
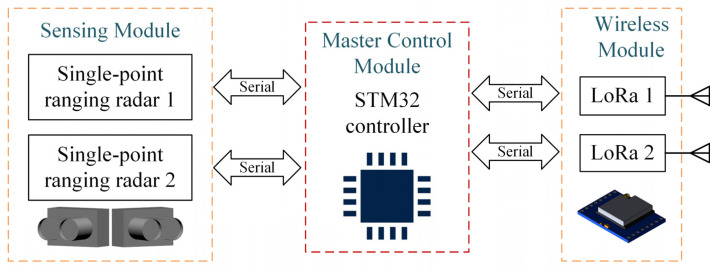
Sensor system architecture.

To simplify the description of the roadside vehicle detection system based on single-point laser ranging, the system will be referred to as a “sensor” from here on. The sensor architecture is illustrated in Figure 1, which consists of an STM32-series microcontroller, two LoRa wireless transceivers, and two single-point laser ranging radars of the same model.

In the experiment, the microcontroller, upon being powered on, initiates data sampling by the single-point laser ranging radars at a frequency of 250 Hz. The collected data can be transmitted to a computer either through wireless transceivers or via a serial port. However, in practical applications, the microcontroller performs the vehicle detection task locally. Instead of transmitting raw sampled data, it uploads only the processed vehicle detection results.

The sensor employs an industrial-grade single-point laser ranging radar with a sampling frequency of 1–4500 Hz and a maximum detection range of 40 m. Typically, this type of radar provides only distance measurements once initiated. However, higher-end single-point laser ranging radars can also output signal strength, indicating the target object’s reflectivity. It is crucial to note that measurement accuracy depends on the target’s reflectivity. When reflectivity is within the normal range, the radar detects a proper reflection signal and provides an accurate measurement. However, if the target’s reflectivity is too low, the weak reflection signal results in the radar returning a zero value. Specifically, this study aims to propose a low-cost vehicle detection system that is most suitable for medium-to-long-range single-point laser ranging radars. Therefore, only the distance measurement value is considered for analysis.

Additionally, the sensor utilizes LoRa wireless communication technology. Although LoRa’s transmission rate is not very high, it is sufficient for low-to-medium-traffic road scenarios, where the frequency of uploading vehicle detection results is low. LoRa offers benefits such as low cost, low power consumption, and long-range communication, making it ideal for building medium-to-long-range sensor networks. Notably, the sensor is equipped with two LoRa wireless communication modules. This setup facilitates the rapid establishment of wireless sensor networks for large-scale traffic monitoring, ensures non-interference between the sensor’s uplink and downlink, and enhances communication efficiency.

**Figure 2:**
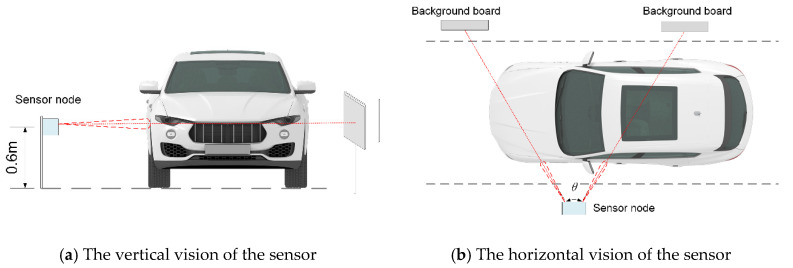
The deployment and detection instructions of the sensor.

Sensor deployment and detection are shown in Figure 2. The sensor is installed at a fixed height above the ground, with the two single-point laser distance sensors set at a fixed angle θ. The central axis of the sensor is perpendicular to the road. Considering the height of most vehicles, it is recommended to install the sensor at an optimal height of 0.6 m to 0.8 m.

The selected single-point laser ranging radar produces zero-value measurements in both the cases of no background and low reflectivity of the target object. To avoid confusion, a background panel can be installed on the opposite side of the road for distinction. If the background value can be detected on the originally deployed section, the background panel is not necessary. It is recommended to choose a white background panel, and its side length $s_{b}$ is determined by the background distance $D_{b}$ and the half-angle β of the laser radar’s field of view. The relationship is defined by the following equation.

(1) $$s_{b}=2D_{b}\tan\beta$$

This ensures that the background panel is appropriately sized to be within the field of view of the laser radar, enabling accurate distinction and measurement.

## 3. Vehicle Detection

### 3.1. Signal Acquisition and Preprocessing

**Figure 3:**
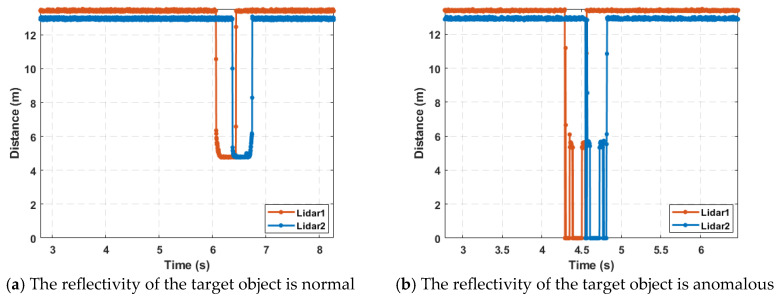
An example of the measured waveform corresponding to a car passing by.

During the signal acquisition process, the disturbance caused by a vehicle passing through the detection area is typically as shown in Figure 3. Figure 3a represents the situation where the reflectivity of the detected area remains good as the target vehicle passes through the detection zone. Figure 3b, on the other hand, represents the situation where the reflectivity of the detected area is anomalous while the target vehicle passes through the detection zone, which is caused by factors such as the vehicle’s structure, material, paint, and posture.

However, as can be seen from Figure 3, regardless of whether the reflectivity of the detected area is good or anomalous when the target vehicle passes through the detection section, the measurements from the single-point laser distance radar consistently show a downward trend. Moreover, there are enough sampling points to determine the vehicle’s current lateral position.

To obtain smoother signals, recursive median filtering [32] is applied to the signal. This method removes part of the impulse noise caused by changes in the detected areas of the vehicle while preserving the edge characteristics of the signal.

(2) $$\left\{\begin{array}{l} y[0]=x[0]\\ y[k]=median(y[\min(0,k-l):k-1],x[k:k+l]),\;l=\left\lfloor \frac{M}{2}\right\rfloor \end{array}\right.$$

Here, x[k] represents the original signal, y[k] represents the filtered signal, M denotes the buffer size, l represents the floor value of half the buffer size, y[min(0,k−l):k−1] indicates the sequence of filtered signals with indices from min(0,k−l) to k−1, and x[k:k+l] denotes the sequence of original signals with indices from k to k+l.

**Figure 4:**
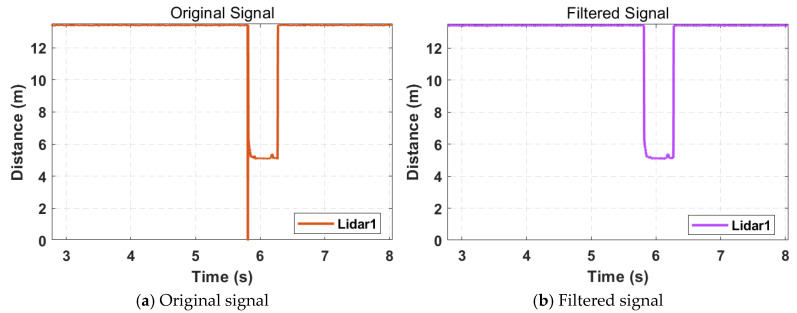
Comparison of original signal and filtered signal.

The filtering effect is shown in Figure 4. Compared with the original signal in Figure 4, the filtered signal has eliminated some of the impulse noise and appears smoother. This improvement facilitates subsequent signal analysis.

### 3.2. Vehicle Detection Algorithm

Vehicle detection is mainly implemented in three steps: the first step involves preliminarily extracting valid waveforms by using an adaptive threshold state machine [33], the second step segments the valid waveforms to identify vehicle occlusion scenarios, and the third step estimates the vehicle’s motion parameters.

#### 3.2.1. Adaptive Threshold State Machine

**Figure 5:**
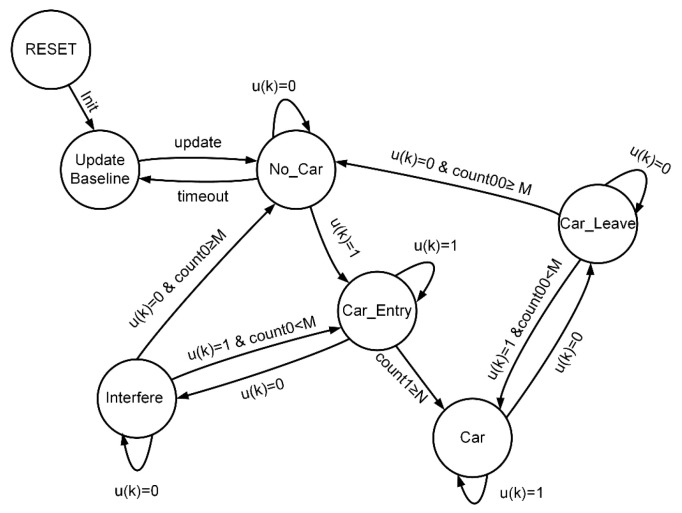
State machine diagram for valid waveform extraction.

As described above, when background values are present, the disturbance caused by a vehicle passing through the detection area typically shows a downward change, which is easy to identify. Based on this characteristic, an adaptive threshold state machine [34] was designed in this study to preliminarily extract the corresponding measurement waveform (valid waveform) of the moving vehicle, as illustrated in Figure 5.

$S_{1}$ (“Baseline Update”): If the system is reset, a baseline update sliding window $W_{b}$ is initiated after successful initialization, with a window size of 5 s. Sampling data are sequentially input into the sliding window. If the baseline update window is full and the range of values is less than the threshold $T_{b}$, the average value is calculated to update the baseline $y_{b}$, and the state transitions to $S_{2}$. If the state transitions from $S_{2}$ to $S_{1}$, the baseline is adaptively updated.

$S_{2}$ (“No Vehicle”): After the baseline is updated, the state transitions to this stage. If $u_{k}=1$, indicating suspected vehicle entry causing occlusion, the state transitions to $S_{3}$ for further confirmation. Otherwise, it checks whether the time without a vehicle exceeds 5 s. If it exceeds 5 s, the state transitions to $S_{1}$. Note that $u_{k}=1$ only when the sampling data are fewer than the threshold $T_{u}$; otherwise, $u_{k}=0$.

$S_{3}$ (“Vehicle Entering”): The vehicle entry counter count1 is started. If the cumulative count reaches the critical value N, the vehicle entry is confirmed, and the state transitions to state $S_{5}$. If $u_{k}=0$, the signal is suspected to include noise data, and the state transitions to $S_{4}$ for confirmation.

$S_{4}$ (“Noise Interference”): In this state, the noise counter count0 is started. If $u_{k}$ continuously equals 0 and the cumulative count reaches the critical value M, noise interference is confirmed, and the state transitions back to $S_{2}$. If $u_{k}=1$ occurs during counting, the state transitions back to $S_{3}$.

$S_{5}$ (“Vehicle Detected”): This state indicates that a vehicle has been successfully detected. If $u_{k}=1$, it suggests that the vehicle has not yet left the detection area. If $u_{k}=0$, it indicates the vehicle may have left, and the state transitions to $S_{6}$ for further confirmation.

$S_{6}$ (“Vehicle Exiting”): The vehicle exit counter count00 is activated. If $u_{k}$ continuously equals 0 and the cumulative count reaches the critical value M, it is confirmed that the vehicle has left, and the state returns to $S_{2}$. If $u_{k}=1$ occurs during counting, the state returns to $S_{5}$.

Here, it is necessary to clarify how to choose the appropriate baseline update threshold $T_{b}$ and vehicle detection threshold $T_{u}$, as well as how to determine the appropriate critical values N and M. First, the baseline update threshold $T_{b}$ is selected based on the measurement error characteristics of the single-point laser rangefinder, and the vehicle detection threshold $T_{u}$ is determined by the number of lanes and the lane width. Secondly, the critical value N is given by Formula (3).

(3) $$N=\frac{3.6\times L_{\min}}{V}\cdot f$$

In the formula, $L_{\min}$ represents the minimum vehicle length to be detected (in meters), V is the average speed of the vehicle (in km/h), and f is the sampling frequency of the single-point laser rangefinder (in Hz).

The critical value M can be determined by the vehicle headway distance within the same lane, as shown in Equation (4).

(4) $$M=\frac{3.6\times L_{f}}{V}\cdot f$$

In the formula, $L_{f}$ represents the vehicle headway distance within the same lane.

Specifically, to respond more quickly to detection, $L_{\min}$ can generally be taken as a value smaller than the minimum vehicle length to be detected, such as half of $L_{\min}$. Similarly, $L_{f}$ can be taken as a value smaller than the actual vehicle headway distance within the same lane. For example, under non-congested conditions, the vehicle headway distance is generally greater than 5 m, in which case $L_{f}$ can be taken as 5.

**Figure 6:**
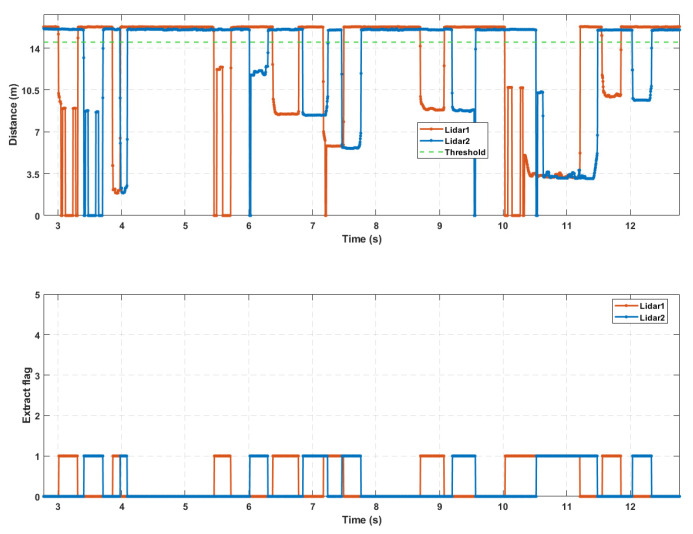
Valid waveform extraction.

By running the above state machine, the valid waveforms caused by the passing vehicles can be preliminarily extracted, denoted by Z, as shown in Figure 6. This is the result of extracting the valid waveforms from 5 vehicles passing through a four-lane detection area with a single-point laser radar. During vehicle detection, when vehicles from different lanes arrive in the detection area at nearly the same time or when there is heavy traffic causing occlusion between vehicles, the valid waveforms may overlap. Therefore, further segmentation and fusion processing of the preliminarily extracted valid waveforms is required.

#### 3.2.2. Segmentation and Fusion Processing

**Figure 7:**
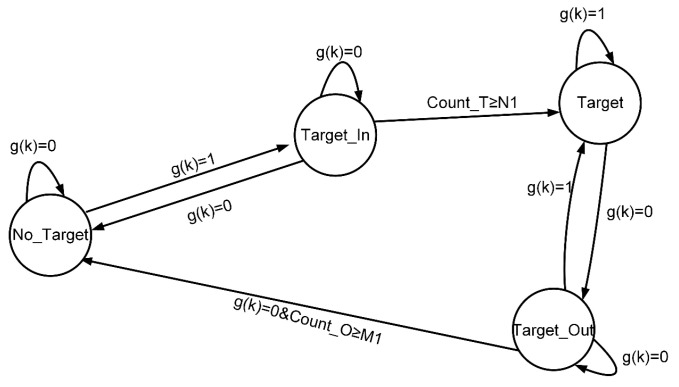
Split state machine.

To address the issue of overlapping valid waveforms, the solution is to establish multiple independent segmentation state machines based on the number of detection lanes. These state machines segment the valid waveforms and extract their features, preparing for the next step of distinguishing vehicles with occlusion in the detection area, as shown in Figure 7.

First, multiple independent segmentation state machines are established based on the number of detection lanes, including the valid data (lane data) segmentation state machine and the anomaly data segmentation state machine. Then, the valid waveform data $z\left(k\right)$ are input sequentially and converted from $z\left(k\right)$ to $g\left(k\right)$ by using the following formula:

(5) $$l_{i}=L_{n}-\left\lfloor \frac{z(k)\cdot \cos\frac{\theta }{2}-d_{I}}{L_{n}}\right\rfloor$$

(6) $$\left\{\begin{array}{l} l_{i}=l_{f}\Rightarrow g(k)=1\\ l_{i}\neq l_{f}\Rightarrow g(k)=0 \end{array}\right.$$

In the formula, $l_{i}$ represents the estimated lane, $L_{n}$ is the number of lanes, θ is the angle between the two laser rangefinders, $d_{I}$ is the installation distance of the sensor from the nearest lane line, and $l_{f}$ represents the lane corresponding to the segmentation state machine. To standardize the operation of each independent segmentation state machine, the corresponding lane for anomalous data (zero value) can be set as $L_{n}+1$.

The counter threshold values, N1 and M1, for the effective data segmentation state machine can be adjusted to appropriate values according to the following formula:

(7) $$C=\frac{3.6\times d_{i}}{V}\cdot f$$

In the formula, C is the critical value, $d_{i}$ is the minimum detection length, and the meanings of the other symbols are the same as those in Equation (3). Generally speaking, the smaller C is, the higher the sensitivity is. As for the counter threshold values N1 and M1 in the anomaly data segmentation state machine, they are set to 2 and 2, respectively. This is because, in practical detection, the length of the anomaly data segments is generally short.

**Figure 8:**
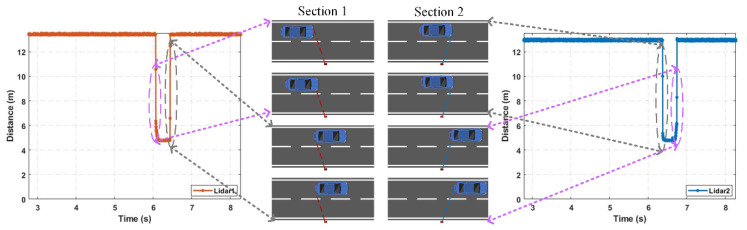
Trailing data.

In addition, when a vehicle passes through the detection area, trailing data may be generated at the edges, as shown in Figure 8. To ensure accuracy in detection, data segments that are short in length and have a large range should be discarded.

While running the segmentation state machine, the minimum sampling value and start and end indices of each data segment are recorded. After the preliminarily extracted valid waveforms are segmented, the anomalous data segments and valid data segments are fused. The specific fusion approach is to compare the time distance between the anomalous data segment and the adjacent valid data segments. The anomalous data segment is then assigned to the nearest valid data segment, and the start and end indices of the valid data segment are adjusted accordingly.

**Figure 9:**
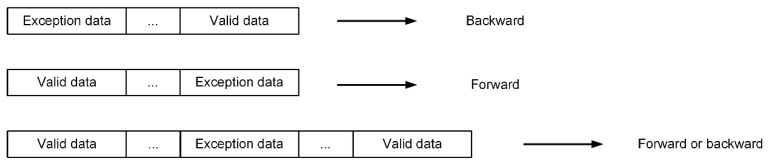
Exception data fusion.

Based on the relative order of the anomalous data segment and the adjacent valid data segments, the fusion direction of the anomalous data segment can be classified into backward fusion, forward fusion, and bidirectional fusion, as shown in Figure 9.

**Figure 10:**
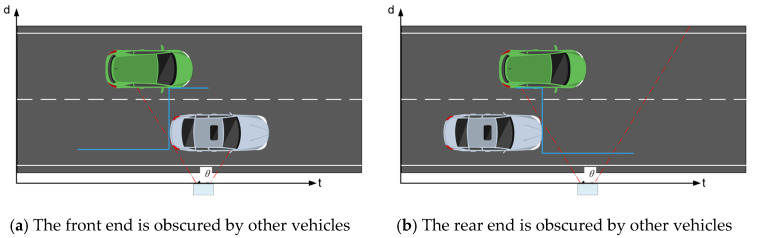
Basic types of vehicle occlusion.

In detection, occlusion is generally divided into two basic types: front occlusion and rear occlusion. The distinction between the two lies in whether the occlusion edge is rising or falling. Most complex cases can be formed by combining these two basic types, as shown in Figure 10. To obtain the accurate timing of vehicle passage through the detection area for speed estimation, vehicle occlusions are further categorized into four situations: no occlusion, front occlusion, rear occlusion, and front-and-rear occlusion. These are represented as 0, 1, 2, and 3, respectively.

Based on the results of anomalous data fusion, further fusion and segmentation of the valid data segments are performed. The basic approach is as follows: For data segments within the same lane, if the time gap between adjacent data segments is smaller than the critical value $P_{f}$, they are fused. Otherwise, they are recognized as separate new incoming vehicles. For data segments of different lanes, if the absolute difference between the non-zero minimum values of adjacent data segments exceeds the threshold $T_{e}$, then the time gap between adjacent data segments is evaluated. If the time gap exceeds the critical value $P_{o}$, they are recognized as separate new incoming vehicles. Otherwise, they are recognized as occlusion, and the type of occlusion between the current and previous vehicles is determined. If the absolute difference between the non-zero minimum values of the data segments is smaller than the threshold $T_{e}$, it is considered that the vehicle may be changing lanes, and the segments are merged with the adjacent data segments. The pseudocode for the valid data segment fusion and segmentation algorithm is shown in the Algorithm 1.

Here, the threshold $T_{e}$ is the sum of the minimum vehicle width maintained during parallel driving and the lateral safety distance. The time gap critical value $P_{f}$ is determined based on the headway distance of vehicles within the same lane, vehicle structural characteristics, and speed, as shown in Equation (8). This is because certain parts of the bottom trusses of some large vehicles are positioned higher off the ground, causing the single-point laser ranging radar to directly detect the background, resulting in a time gap between valid waveforms. Introducing this parameter can effectively determine whether adjacent data segments within the same lane belong to the waveform of the same vehicle. The introduction of the time gap critical value is to avoid the influence of trailing data, enabling a clearer capture of the waveform edges of vehicles in different lanes. The value of this parameter is given by Equation (9), and it is determined by the number of trailing data points and the sampling frequency. In this study, the number of trailing data points is generally between five and seven and is determined by the performance of the single-point laser ranging radar.

(8) $$P_{f}=\frac{3.6\times L_{gap}}{V}$$

In the formula, $L_{gap}$ represents the length of the part of the vehicle that cannot be detected by the single-point laser ranging radar during the detection of the same vehicle. It is recommended that this parameter be slightly larger but still smaller than the vehicle following distance. V represents the vehicle speed.

(9) $$P_{o}=\frac{Num_{Tail}}{f}$$

In the formula, $Num_{Tail}$ represents the number of trailing data points. f represents the sampling frequency.
**Algorithm 1.** Valid data segment fusion and partitioning algorithmInput: valid_data_segments, start_indexs, end_indexs Output: cars_list, front_edges, back_edges, occ_type_list 1. for each data segment *i*: 2.  if cars_list is empty: 3.    create a new car segment 4.  else: 5.    let *j* be the last car segment, and *l* be the second-to-last segment (if it exists) 6.    if the minimum difference between segment *i* and segment *l* < $T_{e}$ and time difference < $P_{f}$: 7.      fuse with segment *l*
8.    else if the minimum difference between segment *i* and segment *j* < $T_{e}$ and time difference < $P_{f}$: 9.      fuse with segment *j*
10.  else if the time difference < $P_{o}$: 11.    mark as occlusion 12.  else: 13.    create a new car segment End

**Figure 11:**
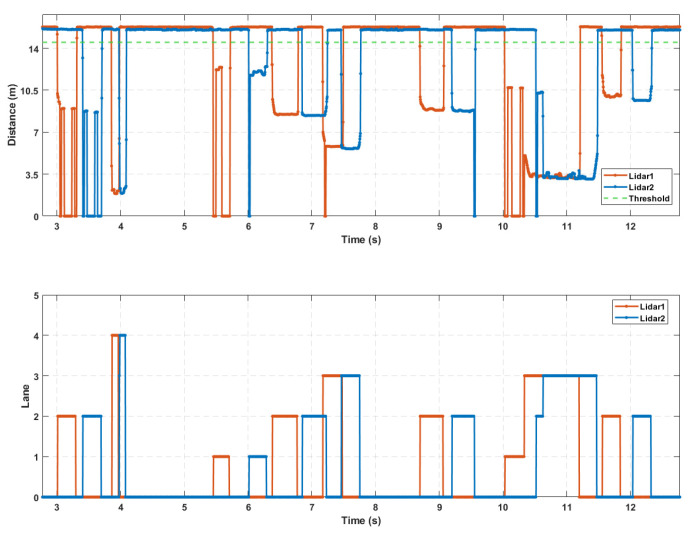
Segmentation fusion result.

The final lane-based extraction result is shown in Figure 11, and as can be seen from the figure, after the series of segmentation and fusion operations mentioned above, the issue of concatenation in the initially extracted valid waveforms has been effectively resolved.

#### 3.2.3. Vehicle Motion Parameter Estimation

**Figure 12:**
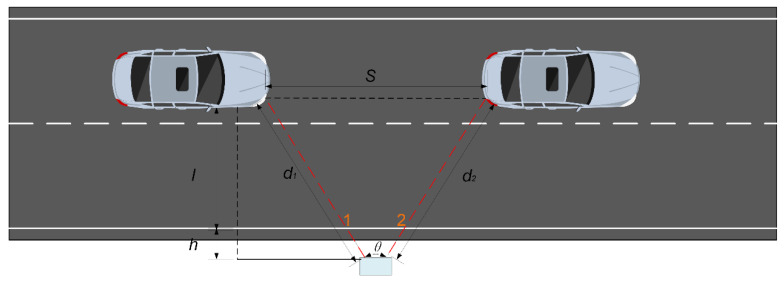
Illustration of vehicle parameter estimation.

As shown in Figure 12, the vehicle parameter estimation formulas are as follows.

The traffic flow is defined as the total number of vehicles passing through the cross-section of a lane in a unit of time. The calculation formula is as follows:

(10) $$Q=\frac{N_{c}}{t_{u}}$$

In the formula, Q represents the traffic flow, and $N_{c}$ is the total number of vehicles passing through the lane cross-section within time $t_{u}$. It is calculated by rounding up the average of the number of vehicles detected in detection section 1 and detection section 2.

When a vehicle moves from detection section 1 to detection section 2, assuming that the lateral distance of the vehicle remains approximately unchanged, by applying the law of cosines, we have

(11) $$S=\sqrt{{d_{1}}^{2}+{d_{2}}^{2}-2d_{1}d_{2}\cos\theta }$$

In the formula, S represents the driving distance, $d_{1}$ is the minimum distance value when the vehicle passes through detection section 1, and $d_{2}$ is the minimum distance value when the vehicle passes through detection section 2.

Vehicle speed is approximately estimated by the average speed of the vehicle as it passes through detection section 1 and detection section 2. The estimation formula is as follows:

(12) $$v=\frac{S}{\Delta t}$$

In this formula, Δt represents the time difference between the vehicle passing through detection section 1 and detection section 2.

The estimation of Δt is determined by the vehicle occlusion-type combination between detection section 1 and detection section 2, as shown in the table below (Table 2). Vehicle speed can only be estimated when the time difference is calculable.

Then, if the vehicle occlusion type in either detection section 1 or detection section 2 is “no occlusion” and the vehicle speed is estimable, the vehicle length can be estimated by using the following formula:

(13) $$l=v\times (t_{li}-t_{ai})$$

In the formula, l represents the estimated vehicle length, i denotes the detection section with no occlusion, $t_{ai}$ and $t_{li}$ represent the entry and exit times of the vehicle in detection section i, and v is the estimated vehicle speed.

## 4. Experiments and Result Analysis

This paper focuses on vehicle detection on low-to-medium-traffic roads. A low-traffic segment and a high-traffic segment were selected as test scenarios. The aim is to verify the feasibility and effectiveness of the roadside vehicle detection system based on single-point laser ranging radar.

### 4.1. Subsection

**Figure 13:**
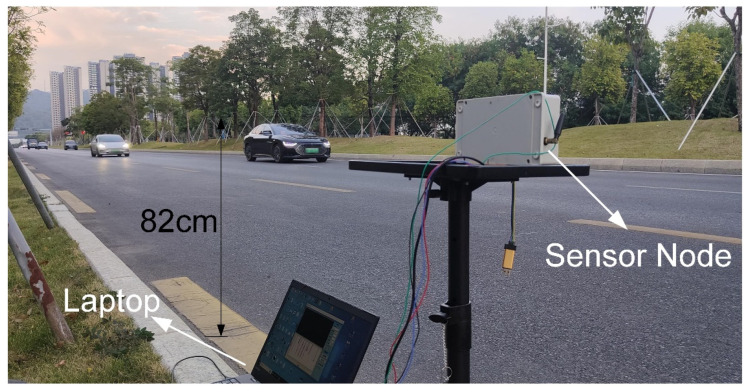
The scene of the low-traffic road experiment.

The low-traffic road section consists of 6 lanes, with the driving directions being from east to west and from west to east, separated by a median strip. There were 3 lanes on each side, with a lane width of 3.5 m. Experimental testing started at 16:00, with clear weather. The detection area focused on the 3 lanes along the east-to-west direction, as shown in Figure 13. Upstream of the road section, there was a traffic light, and after each green light was released, the vehicle flow reached the detection area and completely left within about 23 s. The number of vehicles ranged from 7 to 11, corresponding to a traffic flow of approximately 1100 vehicles/h to 1700 vehicles/h. A handheld radar gun was employed to measure vehicle speeds, which varied between 50 km/h and 70 km/h. Additionally, a mobile phone camera was used to record the lanes in which the vehicles were traveling. The main parameters of the detection algorithm are listed in Table 3.

The traffic parameters measured in the low-traffic road section included the number of vehicles, vehicle speed, and the lane in which the vehicles were traveling. The measurement results are shown in Table 4. The measurement duration was approximately 20 min, with 11 green light releases. A total of 91 vehicles were manually observed, and 92 vehicles were detected, with 91 vehicles having calculable speeds, resulting in an accuracy rate of about 98.9%. The discrepancy was due to a large vehicle with a high chassis and a large gap in the middle, which caused errors during the fusion of effective data segments.

For the detected vehicles, the correct lane identification count was 91, resulting in an accuracy rate of 98.9%. Additionally, the total number of vehicles for which speed could be calculated as 91, and the number of vehicles for which vehicle length could also be estimated as 91. The handheld radar gun recorded the speed of 53 vehicles in the first 6 green light releases. Compared with the speed measured by the handheld radar, the estimated vehicle speed had an average error of 4%, with the maximum error being 10.2%.

### 4.2. High-Traffic Road Section

**Figure 14:**
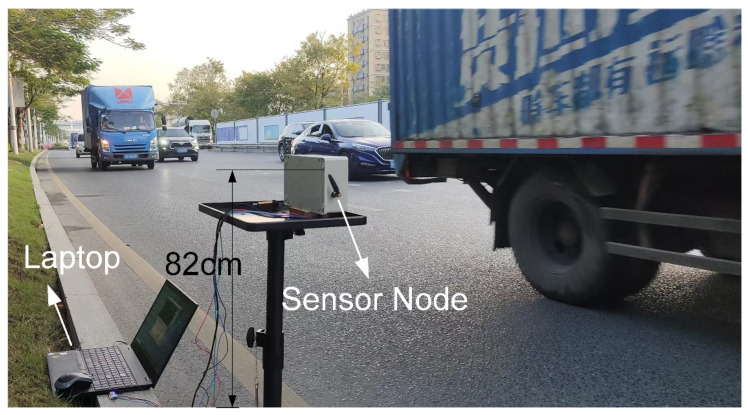
The scene of the high-traffic road experiment.

The high-traffic road section consisted of an 8-lane road, with two directions of travel: from south to north and from north to south. The lanes were separated by a median strip, with 4 lanes on each side, and each lane had a width of 3.5 m. Experimental testing was conducted starting at 17:10 on a sunny day, with the detection area being the 4 lanes along the north-to-south direction, as shown in Figure 14. Upstream of the road section, there were traffic lights. After each green light release, the traffic flow entered and completely exited the detection area in approximately 40 s, with the number of vehicles ranging from 35 to 43 cars. This resulted in a traffic flow rate of approximately 3100 vehicles per hour to 3700 vehicles per hour. Vehicle speeds, ranging from 35 km/h to 55 km/h, were measured by using a handheld radar gun. Additionally, a smartphone camera was used to record the lanes in which the vehicles were traveling. The primary parameters for the detection algorithm are shown in Table 5.

In the high-traffic road section, due to the decreased accuracy of the handheld radar gun in multi-vehicle scenarios and the large volume of traffic, it was difficult to record the speed of each individual vehicle. Therefore, the measured traffic parameters included the number of vehicles and the lane in which each vehicle was traveling, with the measurement results shown in Table 6. The measurement duration was approximately 20 min, with 9 green light releases, during which manual observation recorded 337 vehicles, while 329 vehicles were detected, yielding an accuracy of 97.6%. For the detected vehicles, the number of correctly identified lanes was 325, resulting in an accuracy of 98.7%. Additionally, 319 vehicles had calculable speeds, and 307 vehicles had estimable lengths.

**Figure 15:**
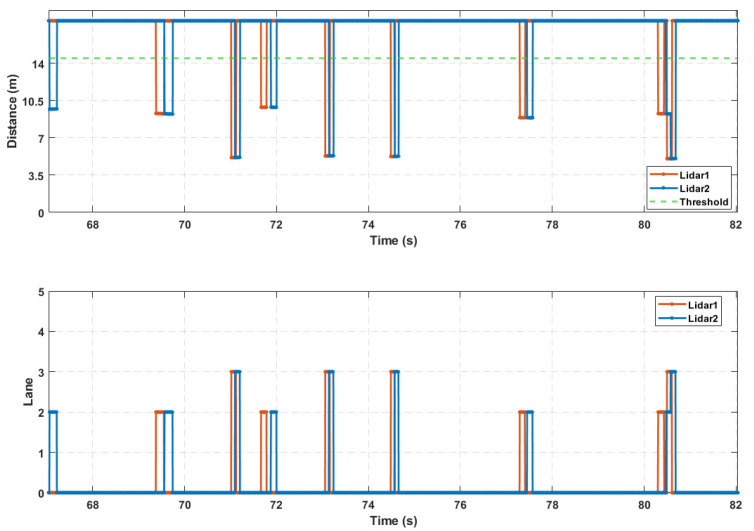
Sensor node acquisition and waveform extraction results.

Due to the interference from vehicles in adjacent lanes during the measurement of individual vehicle speeds, the accuracy of the handheld radar decreased in dense traffic scenarios, preventing an effective comparison with the detection results. Therefore, to further validate the performance of the roadside vehicle detection system based on single-point laser ranging radar in vehicle measurement and length estimation in high-traffic conditions, this study used the real-world HighD dataset [35] as the input for the microscopic traffic flow of the road section. Python 3.7 was used for simulation sensor node sampling at a sampling frequency of 250 Hz. A random selection of 15 s of simulation sampling results and final waveform extraction results are displayed, as shown in Figure 15. Finally, the detection results from the simulation data collection are presented in Table 7, with the estimated vehicle speed error having an average deviation of approximately 1.1% and the estimated vehicle length error having an average deviation of approximately 3.6%. However, in actual detection, the single-point laser ranging radar sampling exhibits tailing effects, and the influence of anomalous reflectivity from the monitored sections can slightly increase the estimation errors for vehicle speed and length.

In summary, the proposed vehicle comprehensive information detection technology based on single-point laser ranging demonstrates significant potential and strong performance in practical applications. In low-traffic road scenarios, where mutual occlusion between vehicles is less common, the system can accurately obtain traffic information such as traffic flow, vehicle speed, and driving lanes. In high-traffic road scenarios, mutual occlusion between vehicles occurs more frequently, leading to issues such as missed detections and inability to estimate vehicle speed. However, the system still achieves an accuracy rate of no less than 95% in traffic flow monitoring and lane identification. Additionally, the proportion of vehicles for which speed can be estimated reaches 94%, and the proportion of vehicles for which length can be estimated reaches 91%.

## 5. Discussion

**Figure 16:**
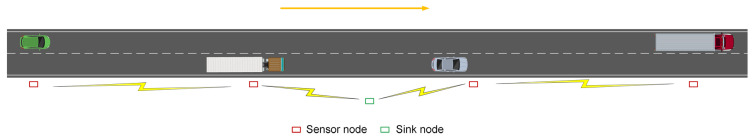
Deployment schematic of multi-point distributed sensor network.

As described above, when balancing cost and efficiency, adopting a sensor network solution for large-scale traffic monitoring on intelligent roads is more appropriate. The vehicle detection system based on single-point laser ranging radar is equipped with two built-in LoRa wireless communication modules for sensor node data transmission. This design allows the system to be easily expanded into a multi-point distributed sensor network. The deployment schematic is shown in Figure 16.

In the team’s previous research study [35], a method was proposed for achieving holistic perception of vehicle trajectories on highways by using low-cost laser sensing devices distributed along roadsides. This method obtains cross-sectional information of vehicle trajectories via a roadside distributed sensor network, including longitudinal position, lateral position, speed, length, lane number, and timestamps. Then, by using conditional matching, the information from adjacent sections is correlated to generate vehicle trajectory point sequences, and the complete vehicle trajectory is reconstructed by using cubic spline interpolation. The single-point laser ranging vehicle detection system proposed in this paper serves as a foundational sensor node for the described method. It captures cross-sectional vehicle trajectory data, including longitudinal position (sensor node installation location), lateral position, lane number, speed, vehicle length, and timestamps, enabling more detailed traffic perception.

**Figure 17:**
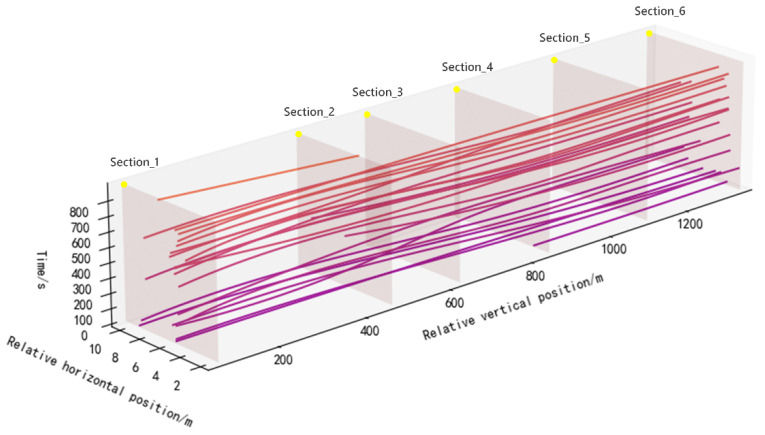
Vehicle trajectory reconstruction based on cross-section information association. The lines in different colors in the coordinate system represent the reconstructed trajectories of different vehicles.

Currently, the team has deployed 22 such sensor nodes along the bridge–tunnel section of a mountainous highway, establishing a multi-point distributed sensor network covering a range of 4.6 km for long-term data collection. The peak hourly vehicle flow on this section is approximately 217 vehicles per hour. A 1.5 km segment of this section was selected as the experimental scenario, where vehicle speed, type, lane, and timestamps were recorded at each sensor node by using the vehicle license plate matching method. These were then compared with the distributed sensor detection data and association results. According to the data recorded by the license plate matching method, the accuracy of matching and associating detection data between adjacent sensor nodes reached 98.4%. A 10 min reconstructed vehicle trajectory was selected for display, as shown in Figure 17.

## 6. Conclusions

### 6.1. Achievements and Limitations

This paper proposes a vehicle comprehensive information detection technology based on single-point laser ranging, and on this basis, a corresponding roadside vehicle detection system is developed and designed. The system features low cost, easy installation and maintenance, and good scalability, making it effective for traffic flow detection and estimation of multiple vehicle operation parameters. Through steps such as data preprocessing, the preliminary extraction of valid waveforms, valid waveform segmentation and fusion processing, and vehicle parameter estimation, this technology can accurately obtain information such as the speed, length, lateral position, and lane of passing vehicles. Experiments conducted on road sections with varying traffic flow demonstrate that the technology performs excellently in low-traffic, multi-lane scenarios and still maintains high detection accuracy in high-traffic, multi-lane scenarios. In addition, the Discussion section presents some work related to large-scale traffic monitoring using this technology. By constructing a multi-point distributed sensor network and combining technologies such as multi-vehicle tracking and vehicle trajectory reconstruction, more refined traffic perception can be achieved. It indicates that this technology has great potential for application.

However, the current detection method remains relatively complex, with some shortcomings in terms of real-time vehicle detection. Additionally, the method requires the provision of a measurement background in real-world scenarios, which is not applicable to many bidirectional roads in urban areas. Of course, this can be adapted by adjusting the detection algorithm, though the current detection algorithm does not yet meet the necessary requirements. Moreover, as the traffic flow increases, the phenomenon of vehicle occlusion intensifies, which leads to a decline in the system’s recognition performance. Therefore, the primary application scenarios for this technology are secondary roads with medium- and low-traffic flow, mountainous highways, and suburban roads. It is not suitable for road sections with high-traffic flow and more complex traffic compositions. Furthermore, in practical applications, single-point laser ranging radars inevitably face the impact of adverse weather conditions such as heavy rain, snow, and fog, which may result in inaccurate measurements under low-visibility conditions.

### 6.2. Future Research Directions

Future research will focus on two main directions: first, optimizing the vehicle detection system based on single-point laser ranging; second, applying the system to large-scale traffic monitoring on medium- and low-traffic flow road sections.

In terms of optimizing the vehicle detection system, the following directions can be pursued: First, the vehicle detection algorithm can be optimized to improve real-time performance in multi-lane detection. In essence, the vehicle detection method proposed in this paper initially extracts valid waveforms based on an adaptive threshold state machine, which are waveforms of multi-lane traffic segments. Therefore, the waveforms need to be segmented and fused to extract the valid waveform of a single vehicle. In theory, these two steps can be further merged to improve processing efficiency. Second, the system’s ability to recognize occlusion should be enhanced to improve accuracy in vehicle detection. This can be achieved through machine learning methods, using multi-classification algorithms to directly extract the valid waveforms of individual vehicles and recognize occlusion types. However, this method depends on labeled datasets during the training process, and an experimental platform would need to be set up for data collection. Additionally, consideration can be given to using laser radar that returns target object reflection intensity information. By analyzing the variation in reflection intensity, vehicle occlusion can be identified. Moreover, combining vehicle reflection intensity waveforms and vehicle length information could further refine vehicle-type classification. However, the use of such single-point laser ranging radar may increase the system’s cost, as such radars are typically more expensive.

In terms of large-scale traffic monitoring, research will be extended from a single sensor node to a multi-point distributed sensor network, combined with multi-target tracking and vehicle trajectory reconstruction technologies, to achieve large-scale, long-distance vehicle trajectory holographic sensing. This direction involves several aspects, including communication networks, multi-sensor fusion, and multi-target tracking. First, in terms of communication networks, it is essential to design a low-power, low-latency communication scheme suitable for multi-point distributed sensor networks. Second, in terms of multi-sensor fusion, the coverage distance of distributed sensor networks is considerable, involving the fusion of data from both same-type sensors and different-type sensors. The optimal layout scheme, data synchronization, spatiotemporal alignment, and information fusion algorithm design are all current research hotspots. Finally, in multi-target tracking, the continuous estimation of the operating status of multiple vehicles based on detection information from multiple sections requires further research. Additionally, on this basis, vehicle trajectory reconstruction and prediction tasks can also be explored.

## Figures and Tables

**Table 1:** Comparison of vehicle detection technologies.

Detection Technology Types	Detection Indicators and Performance	Detection Range		Deployment Cost	Installation Method
		Coverage	Lane Count		
Inductive loop detection	⬤ Indicators: Vehicle presence Vehicle speed Lane occupancy status Vehicle type Driving lane ⬤ Precision: High	▲	1	Low	Buried installation
Geomagnetic detection	⬤ Indicators: Vehicle presence Vehicle speed Vehicle type Driving lane ⬤ Precision: High	▲	1	Low	Buried installation
Ultrasonic detection	⬤ Indicators: Vehicle presence Vehicle speed Vehicle type Driving lane ⬤ Precision: Medium	8–20 m	1	Medium	Pavement installation
Video detection	⬤ Indicators: Vehicle presence Vehicle speed Vehicle type Driving lane ⬤ Precision: Medium	*	*	High	Pavement installation or roadside installation
LiDAR detection	⬤ Indicators: Vehicle presence Vehicle speed Vehicle type Vehicle position ⬤ Precision: High	≤180 m	≤8	High	Pavement installation or roadside installation
Dual single-point laser radar detection (this study)	⬤ Indicators: Vehicle presence Vehicle speed Vehicle type Vehicle position ⬤ Precision: Medium	▲	≥1	Low	Roadside installation

(▲ indicates that the coverage area is the section at the installation location of the sensor node. * indicates that the detection range is highly affected by the product type).

**Table 2:** Vehicle travel time difference calculation.

Occlusion-Type Assemble	Δt
{0, 0}/{0, 1}/{1, 1}	$\Delta t=t_{l1}-t_{l2}$
{0, 2}/{2, 2}	$\Delta t=t_{a1}-t_{a2}$
{1, 2}	Unable to be calculated
{3, -}	Unable to be calculated

**Table 3:** Parameter settings (low-traffic road).

Parameter	Symbol	Value
The angle between two laser ranging radars	θ	40°
Sensor node installation distance	-	0.2 m
Baseline update threshold	$T_{b}$	0.5 m
Vehicle detection threshold	$T_{u}$	11 m
Vehicle entry counter critical value	N	40
Vehicle leave counter critical value	M	80
Valid data segment fusion threshold	$T_{e}$	2.2 m
Time gap critical value	$P_{f}$	0.32 s
Time gap critical value	$P_{o}$	0.028 s

**Table 4:** Low-traffic road experiment results.

	Low-Traffic Road		
	Observation Results	Detection Results	Correct %
Vehicle count	91	92	98.9
Vehicles with speed		91	100
Vehicles with length		91	100
Lane identification		92	98.9
	Max Error %	Min Error %	Average Error %
Speed error	0.102	0.001	0.040

**Table 5:** Parameter settings (high-traffic road).

Parameter	Symbol	Value
The angle between two laser ranging radars	θ	40°
Sensor node installation distance	-	0.2 m
Baseline update threshold	$T_{b}$	0.5 m
Vehicle detection threshold	$T_{u}$	14 m
Vehicle entry counter critical value	N	25
Vehicle leave counter critical value	M	70
Valid data segment fusion threshold	$T_{e}$	2.2 m
Time gap critical value	$P_{f}$	0.28 s
Time gap critical value	$P_{o}$	0.028 s

**Table 6:** Low-traffic road experiment results.

	High-Traffic Road (Actual Measurement)		
	Observation Results	Detection Results	Correct %
Vehicle count	337	329	97.6
Vehicles with speed		319	94.7
Vehicles with length		307	91.1
Lane identification		325	98.7

**Table 7:** High-traffic road experiment results (simulation measurement t).

	High-Traffic Road (Simulation Measurement)		
	Observation Results	Detection Results	Correct %
Vehicle count	306	295	96.4
Vehicles with speed		288	94.1
Vehicles with length		280	91.5
Lane identification		295	96.4
	Max Error %	Min Error %	Average Error %
Speed error	0.055	0.001	0.011
Length error	0.154	0.001	0.036

## Data Availability

The data are not publicly available due to restrictions on privacy.
